# Increased dopamine release after working-memory updating training: Neurochemical correlates of transfer

**DOI:** 10.1038/s41598-017-07577-y

**Published:** 2017-08-02

**Authors:** Lars Bäckman, Otto Waris, Jarkko Johansson, Micael Andersson, Juha O. Rinne, Kati Alakurtti, Anna Soveri, Matti Laine, Lars Nyberg

**Affiliations:** 1 Aging Research Center, Karolinska Institute, Stockholm, Sweden; 2 Department of Psychology, Åbo Akademi University, Turku, Finland; 3 Turku PET Center, University of Turku and Turku University Hospital, Turku, Finland; 4 Division of Clinical Neurosciences, Turku University Hospital, Turku, Finland; 5 Department of Radiation Sciences, Umeå University, Umeå, Sweden; 6 Department of Integrative Medical Biology, Umeå University, Umeå, Sweden

## Abstract

Previous work demonstrates that working-memory (WM) updating training results in improved performance on a letter-memory criterion task, transfers to an untrained n-back task, and increases striatal dopamine (DA) activity during the criterion task. Here, we sought to replicate and extend these findings by also examining neurochemical correlates of transfer. Four positron emission tomography (PET) scans using the radioligand raclopride were performed. Two of these assessed DAD2 binding (letter memory; n-back) before 5 weeks of updating training, and the same two scans were performed post training. Key findings were (a) pronounced training-related behavioral gains in the letter-memory criterion task, (b) altered striatal DAD2 binding potential after training during letter-memory performance, suggesting training-induced increases in DA release, and (c) increased striatal DA activity also during the n-back transfer task after the intervention, but no concomitant behavioral transfer. The fact that the training-related DA alterations during the transfer task were not accompanied by behavioral transfer suggests that increased DA release may be a necessary, but not sufficient, condition for behavioral transfer to occur.

## Introduction

The last 10–15 years have witnessed an explosion of studies examining whether working memory (WM) performance can be enhanced through various training procedures. Different approaches have been employed in this work, ranging from training of specific components of WM such as updating^1–3^, shifting^3, 4^, and inhibition^5^ to composite programs that involve practicing several WM functions^6, 7^. Typically, the training runs for 4–5 weeks, with 3 sessions per week, each lasting for 45–60 min. Several recent reviews^8–11^ give rise to the following conclusions: (a) WM training results in gains on the trained tasks; (b) transfer effects to tasks tapping untrained cognitive domains (e.g., reasoning, intelligence, multitasking) are small or non-existent; and (c) although transfer to untrained WM tasks that share constituent processes with the trained WM tasks may be seen, generalizability to other WM tasks is also quite limited.

Several fMRI studies examining neural correlates of training-related WM gains report increased blood-oxygen-level dependent (BOLD) activity in striatum^1–3, 12^. This is an interesting observation in view of the fact that striatal neurons are thought to serve a gating function in letting new information enter into WM^13–15^. The functional relevance of increased striatal BOLD activity following WM training was demonstrated by Brehmer and colleagues^16^, who reported a strong relationship between the magnitude of training-related increases in striatal BOLD activity and the size of WM improvement post training.

Animal^17, 18^ and human^19, 20^ data reveal a link between BOLD activity and measures of dopamine (DA) release. These associations open up for the possibility that increased striatal BOLD activity after WM training is related to an increased release of DA. To infer DA release during task performance, typically quantified using positron emission tomography (PET) and specific radioligands, two conditions that vary in cognitive demands are contrasted. The idea is that binding of the radioligand to DA receptors should be reduced during the more challenging condition relative to the control condition. This is so because, in the former case, binding of the ligand to receptors competes with binding of endogenous DA to the same receptors to a greater extent than in the control condition. Thus, reduced binding is assumed to reflect an increased release of DA. This displacement principle was initially formulated in the context of pharmacological DA challenges^21^, and evidence for displacement has been observed in both striatal and extrastriatal regions during verbal^22^ and spatial^23, 24^ WM tasks.

Using the same updating training procedure as Dahlin *et al*.^1^, Bäckman *et al*.^19^ conducted a PET study that investigated whether 5 weeks of WM training was associated with increased striatal DA release. In that study, the radioligand raclopride was used to measure DAD2 receptor binding. There were two main PET findings: (a) increased DA release was observed during a trained updating task relative to a low-level control task before training, and (b) WM training resulted in a further increase of DA release. The locus of the DA effects was in left caudate, close by the training-related BOLD changes reported by Dahlin *et al*.^1^ However, as this is the only study documenting increased DA release following WM training, replication of this finding is warranted. Replication of intriguing data is a rare happening in many fields, including cognitive neuroscience. When replication is attempted, a considerable number of findings reported in journals like *Science* and *Nature* cannot be reproduced^25, 26^. In the current work, a major goal was therefore to seek to replicate the Bäckman *et al*.^19^ findings. The training procedure, the criterion task, as well as the PET assessment of DA release were identical to those employed by Bäckman *et al*.^19^.

As noted, transfer effects from this type of WM training to untrained tasks are small or non-existent. However, we observed transfer from WM updating training to an untrained n-back task that shares a similar demand on updating operations^1, 19^. An interesting finding in Dahlin *et al*.^1^ was that transfer effects were also observed in terms of neural activation patterns, as measured by BOLD fMRI. Specifically, increased BOLD activity was seen for both the letter-memory and n-back tasks in an overlapping part of the caudate. That said, the magnitude of the increases in the n-back task was considerably smaller compared to the letter-memory criterion task, both behaviorally and neurally. An additional objective of the present research was to investigate whether updating training would result in increased DA release also in an untrained n-back task, thus providing evidence for a neurochemical correlate of transfer of learning.

## Results

### Letter-memory criterion task

**Figure 1:**
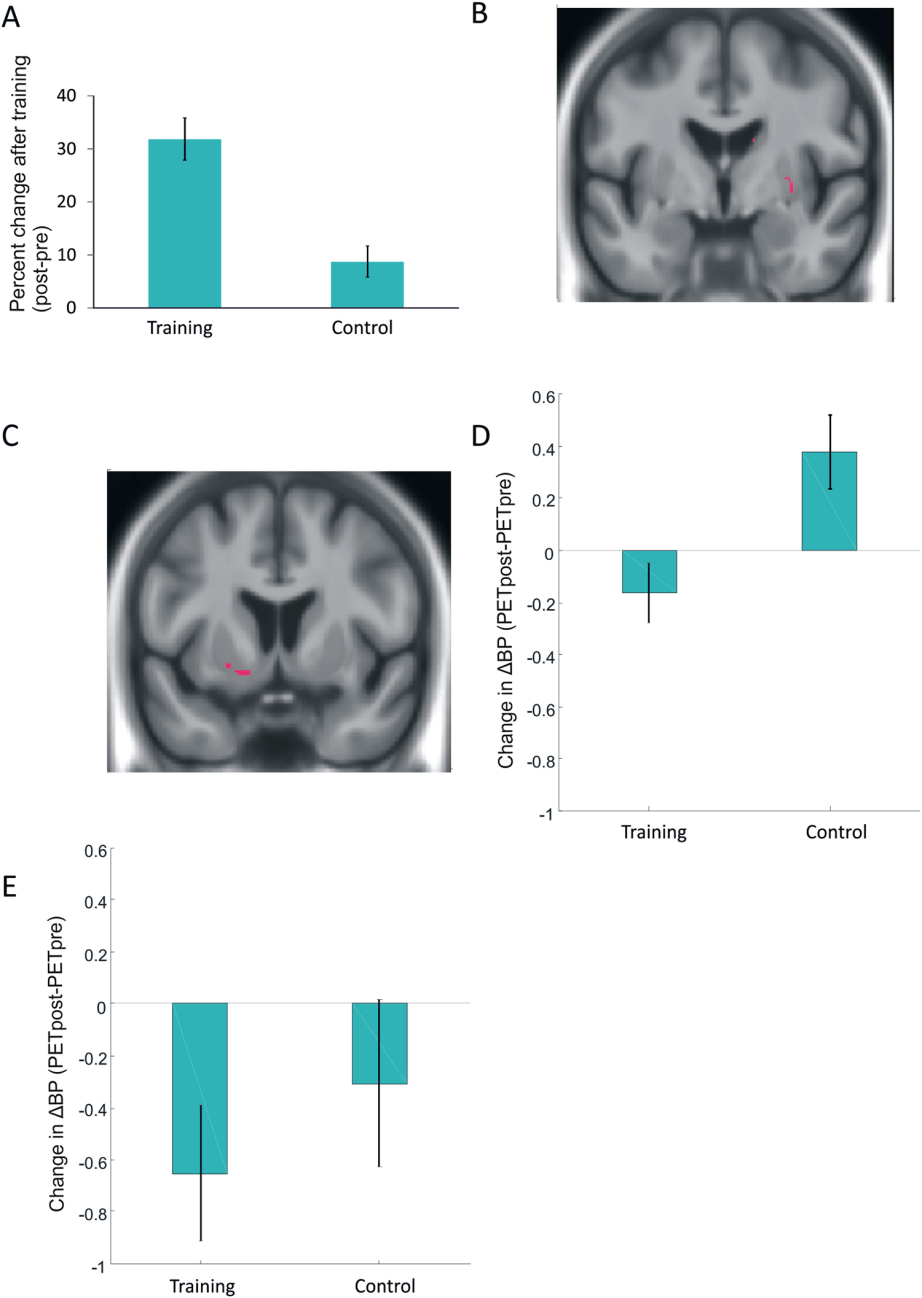
Behavioral and dopamine effects for the letter-memory criterion task. (**A**) Percent pre-post changes in letter memory. (**B**) Lower raclopride binding to striatal D2 receptors during letter memory compared to the control task before training in right striatum, reflecting greater DA release in response to the cognitive challenge (x,y,z = 14,−3,18; 30,−3,−2). (**C**,**D**) A training-induced decrease of raclopride binding to D2 receptors was found in left striatum (−18, 6, −12). (**E**) Training-related changes in raclopride binding to striatal D2 receptors in the peak region observed by Bäckman *et al*.^18^. Error bars are standard errors.

Two participants in the updating training group and one participant in the control group were not included in the letter-memory analysis due to technical problems leading to loss of post-training behavioral data. The main analysis of the letter-memory criterion task revealed a significant group x time interaction (*F*(1, 23) = 24.579, *p* < 0.001, η^2^partial = 0.52; *d* = 2.07), reflecting the fact that the training group showed larger performance gains after training than the control group (Fig. 1A). An identical analysis involving only those participants who were included in the PET analyses yielded the same significant interaction (one participant could not be included in this analysis due to a technical error). Thus, the present behavioral findings replicated exactly those from our two previous studies^1, 19^.

In all analyses of the PET data, we only report effects that exceed 5 voxels to reduce the risk of spurious findings. We first examined a task effect on D2DR BP comparing the letter-memory and baseline tasks before training across all participants with a paired *t* test. We observed decreased D2DR binding during letter memory in bilateral striatum (x,y,z = 14,−3,18; 30,−3,−2, *p* < 0.01; Fig. 1B; x,y,z = −17,21,0; −23,2,11, *p* < 0.05). Critically, an ANOVA on the D2DR BP data yielded a significant group x time interaction in bilateral striatum (x,y,z = 26,−5,8; −18,6,−12, *p* < 0.05). Figures 1C and D highlight this effect for left striatum. This interaction reflected the fact that the trained persons, but not the controls, showed decreased BP after training. In a peak-voxel analysis of a potential training-related change in the exact same region as originally observed (x,y,z = −17,−7,22;^18^), a similar trend of greater reduction of BP in the trained group compared to the control group was observed, although this effect did not reach conventional significance (Fig. 1E; *p* > 0.05).

### Transfer tasks

**Figure 2:**
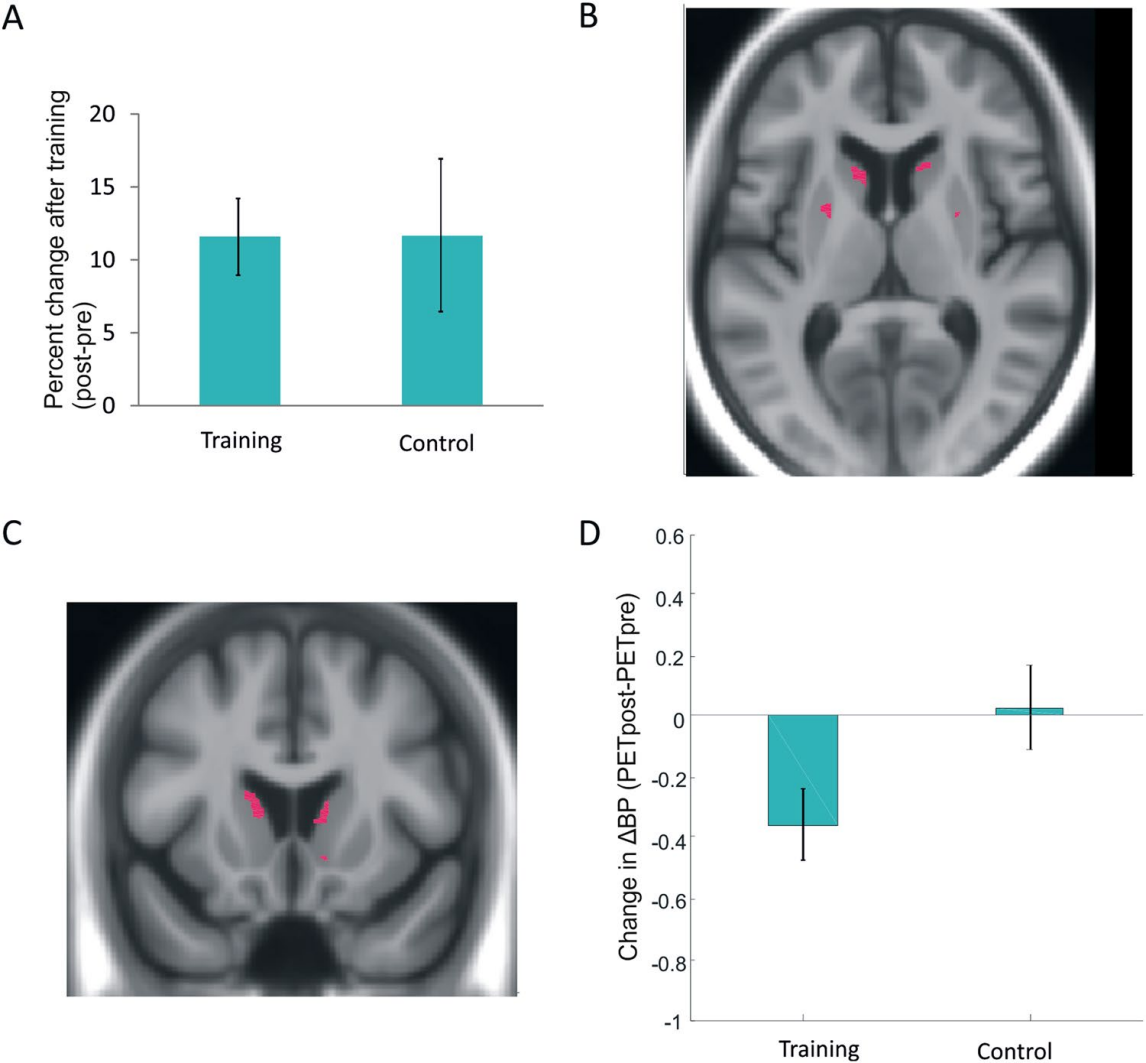
Behavioral and dopamine effects for the n-back transfer task. (**A**) Percent pre-post changes in 3-back. (**B**) Lower raclopride binding to striatal D2 receptors during 3-back compared to 1-back before training in bilateral striatum, reflecting greater DA release in response to the cognitive challenge (x,y,z = 27,0,0; 11,18,6; −23,0,8; −12,15,11). (**C**) Effects in bilateral striatum (x,y,z = 11,14,−9; −17,15,14) showing a training-induced decrease of raclopride binding to D2 receptors during 3-back. (**D**) Bar graph showing a selective training-related reduction in right striatum for trained subjects. Error bars are standard errors.

For several transfer tasks (in-scanner digit n-back and the offline tasks spatial n-back, letter-number sequencing, digit symbol, word recall, and number-letter RT), there were main effects of time (*p*s < 0.05), reflecting general retest effects. However, for the in-scanner 3-back task (Fig. 2A) as well as for all off-line transfer tasks (Table 1), there were no disproportionate time effects as a function of group (*p* > 0.05; *d* = 0.00). Thus, no behavioral transfer effects were observed.

**Table 1:** Group means (*SD*) on the pre- and post-training cognitive measures.

	WM training group		Control group	
	Pre-training	Post-training	Pre-training	Post-training
Letter-memory accuracy %	*n* = 12		*n* = 13	
	51.54 (11.63)	83.33 (31.67)	47.57 (12.87)	56.28 (17.01)
Digit n-back	*n* = 10		*n* = 12	
1-back target accuracy %	91.23 (7.78)	91.58 (7.71)	93.62 (3.05)	89.14 (8.42)
3-back target accuracy %	74.53 (18.24)	86.12 (14.51)	79.44 (20.66)	91.10 (7.30)
Letter-number sequencing	*n* = 14		*n* = 14	
	13.50 (2.79)	14.79 (2.61)	14.00 (3.21)	14.64 (3.84)
Digit span forward	*n* = 13		*n* = 14	
	7.00 (1.23)	7.38 (0.87)	6.43 (1.22)	6.36 (1.15)
Digit span backward	*n* = 14		*n* = 14	
	6.15 (1.21)	6.08 (1.50)	6.36 (1.50)	6.36 (1.28)
Digit symbol	*n* = 14		*n* = 14	
	71.00 (12.27)	77.29 (13.98)	68.07 (11.53)	75.43 (11.65)
Word recall accuracy %	*n* = 14		*n* = 14	
	75.13 (10.54)	80.69 (11.84)	80.16 (15.70)	82.14 (13.33)
Simon task	*n* = 14		*n* = 13	
Congruent RT	413 (45)	381 (49)	430 (58)	400 (42)
Incongruent RT	434 (38)	398 (43)	460 (48)	420 (35)
Congruent accuracy %	99.00 (1.52)	98.86 (1.51)	98.62 (1.89)	98.31 (1.97)
Incongruent accuracy %	96.71 (2.16)	97.71 (2.33)	96.92 (2.78)	97.69 (2.29)
Number-letter task	*n* = 14		*n* = 14	
Mixed task: no-switch RT	680 (104)	599 (78)	767 (116)	642 (97)
Mixed task: switch RT	955 (288)	816 (234)	1115 (310)	866 (193)
Mixed task: no-switch accuracy %	98.94 (2.00)	99.70 (1.14)	98.48 (2.27)	98.78 (1.37)
Mixed task: switch Accuracy %	97.99 (3.15)	97.99 (3.15)	96.21 (3.51)	96.21 (3.50)
Spatial n-back	*n* = 14		*n* = 13	
1-back accuracy in %	96.80 (2.81)	98.31 (2.09)	96.96 (2.91)	98.08 (1.67)
2-back accuracy in %	94.84 (5.15)	97.82 (2.97)	93.27 (5.67)	98.40 (2.53)

However, as with letter-memory, comparing the 3-back and 1-back tasks before training across all participants with a paired *t* test, we observed decreased D2DR binding during 3-back in bilateral striatum (Fig. 2B; x,y,z = 27,0,0; 11,18,6; −23,0,8; −12,15,11, *p* < 0.01). Importantly, a group x time ANOVA on the D2DR BP n-back data yielded a significant interaction in bilateral striatum (Fig. 2C; x,y,z = 11,14, −9; 9,14,8; −17,15,14, *p* < 0.05). The interaction in right striatum reflected the fact that the training group showed decreased BP after training, whereas the control group’s level was stable (Fig. 2D).

## Discussion

The main goal of this study was to replicate the Bäckman *et al*.^19^ finding of increased DA release following WM updating training. First, replicating and extending prior work^19, 22–24^, DAD2 BP was lower during both letter memory and n-back than in the control conditions before training. This pattern indicates increased DA release as a function of the cognitive challenge. A key observation was reduced striatal DAD2 BP during letter memory in the training group at post test, suggesting increased DA release after training. The intervention effect on neurotransmission was accompanied by pronounced training-related behavioral gains in the letter-memory criterion task^1, 19^. Although the DA effect did not overlap with the peak effect obtained in our previous study, both were located in left striatum. Also, a similar trend was seen in the same peak voxel as originally reported^19^.

A unique feature of this study was the use of four PET examinations, allowing us to assess neurochemical effects during an untrained n-back task. Interestingly, our training was associated with increased striatal DA release at the post-training assessment also during the 3-back transfer task. To the best of our knowledge, this is the first demonstration of changes in neurotransmission in relation to transfer of learning. For n-back the peak response was in right striatum, whereas the corresponding effect for letter memory was in left striatum. The reason for the difference in laterality remains unclear, and was not expected from our previous observation of a training-related overlap in left caudate BOLD signal change for letter memory and n-back^1^.

In agreement with several recent meta-analyses and qualitative reviews^8–11^, we found no evidence that updating training transferred to performance on the tasks included in the off-line battery tapping verbal and spatial working memory, motor speed, episodic memory, set shifting, and inhibitory control. Moreover, unlike the findings reported by Dahlin *et al*.^1^ and Bäckman *et al*.^19^, we did not observe behavioral generalization to the 3-back transfer task. The reason thereof may be that the present participants performed at a very high level for 3-back already at the pre-training assessment. The high level may reflect the length (more room for task practice) and the nature of the task (performed during scanning, which likely increases the perceived performance demands compared to an off-line testing situation). Note also that recent meta-analyses indicate that effect sizes for this form of near-transfer are quite small^9–11^. Thus, it is unsurprising that such transfer effects may or may not be observed in a specific study. This is especially true in PET research with relatively small sample sizes and limited statistical power. In view of this concern and even though the measurement scale might not have been sensitive enough to capture behavioral transfer effects, the PET data nevertheless demonstrated DA alterations during the untrained n-back task. The present pattern of results raises the possibility that increased DA release may be a necessary, but not sufficient, condition for behavioral transfer to occur.

The present research extends previous observations that the DA system is plastic. Such plasticity has been observed during both pharmacological and cognitive challenges^18, 20, 21, 27–31^. The current replication of increased striatal DA release during letter memory after WM updating training, and the novel result of a corresponding increase during an untrained 3-back task, provide additional evidence that the DA system is malleable.

## Methods

### Participants

The effective sample included 28 right-handed healthy, non-smoking, and non-medicated Finnish male university students (19–26 years). The participants underwent structural MRI and medical screening. They were randomized into training and control groups (*n* = 14 for each). The two groups were comparable regarding years of education, age, and BDI-II scores, and on all pre-training neuropsychological measures, except for TMT B, *t*(25) = 2.35, *p* < 0.05, where the control group showed significantly worse performance (Table 2).

**Table 2:** Descriptive data on the study groups (means and *SD*).

	WM training group (*n* = 14)	Control group (*n* = 14/13)
Age	22.21 (1.72)	22.79 (1.48)
Years of education	14.21 (1.19)	15.15 (1.45)
BDI-II	2.64 (3.13)	1.79 (1.85)
WAIS-III Vocabulary	52.50 (6.97)	49.86 (7.82)
TMT A	21.64 (5.23)	22.93 (4.57)
TMT B	42.64 (6.79)	50.69 (10.74)
Pattern Comparison	19.61 (3.70)	20.04 (3.20)
Number Copying	51.46 (5.76)	51.04 (6.25)

Note. BDI = Beck Depression Inventory, WAIS = Wechsler Adult Intelligence Scale, TMT = Trail Making Test. Age in years, BDI-II scores, WAIS-III Vocabulary raw scores, TMT completion time in sec, Pattern Comparison and Number Copying raw scores (*SD*s in parentheses). One participant in the control group had missing data on years of education, one participant in the control group had unreliable performance data on the Pattern- Comparison task, and one participant in the control group was an extreme outlier in TMT B (hence, *n = *13 in the control group for these variables).

The study was approved by the Ethics Review Board of the Turku University Hospital District, the methods were carried out in accordance with relevant guidelines and regulations, and written informed consent was obtained from all participants.

### Procedure

We employed a pre-training–intervention–post-training control group design. All participants took part in the pre- and post-training assessments, whereas only participants in the training group received training between these assessments. The training group practiced three times per week (45 min/session) for five weeks. The pre-training assessments included a structural MRI scan, neuropsychological testing, and two consecutive PET scans performed during the same day. The post-training assessments included neuropsychological testing and two PET scans, but no MRI. Time in-between the pre- and post-training assessments was 6–9 weeks.

The training program was a computerized in-house developed Visual Basic program and consisted of the letter memory criterion task and five other updating tasks^1, 19^. Four of the additional training tasks were similar to the letter memory task and involved updating of single items, but to foster generality different kinds of stimuli (i.e. numbers, letters, colors, and spatial locations) were used. In these training tasks, five lists of items were randomly presented and the task was to recall the four last presented items. Across the 5-week training period, list length varied to manipulate level of difficulty and thereby ensure that the training was sufficiently demanding (low level = 4–7 items; medium level = 6–11 items; high level = 5–15 items). Performance was monitored and at the end of each training week, level of difficulty was raised when the participant scored 80% or higher in the letter memory task. All subjects reached the most difficult level by the end of training. The final training task was a keep-track task. This task, too, taxes updating, but is structurally different from the other updating tasks in the training battery. The inclusion of the keep-track task should contribute toward strengthening of a general updating skill. In each trial, 15 words from different semantic categories were presented serially in random order (2.0 s per word) and participants were instructed to place the words into categories (animals, articles of clothing, countries, relatives, sports, professions), indicated by boxes at the bottom of the screen. They had to continuously update their working memory content and remember the last presented word in each category at the end of the presentation. Participants responded by typing the last presented word under each category box when the trial ended.

### PET scanning

The pre- and post-training PET scans were identical: both involved two scans of which one was performed in the morning and the other in the afternoon. The letter-memory task was administered during the morning scan, whereas the digit n-back task was administered during the afternoon scan. The two assessments were separated by a 90-min lunch break.

During the first PET scan, we administered a computerized letter-memory task that taps verbal WM updating. Participants were shown 7–15 letters in a sequence; when a sequence suddenly ended, they were asked to report the last four letters in correct order by pressing buttons corresponding to A, B, C, and D. This task was preceded by a structurally equivalent control task not taxing updating (all letters in a sequence were identical and participants reported that letter). Prior to PET scanning, participants acquainted themselves with the task during a practice session. The PET session started with the control task (5–10 min prior to bolus injection continuing for 55 min post-injection), followed by the letter-memory task (25 min). The sequence began with instructions shown for 7000 ms. Stimulus duration was 2000 ms with a fixation cross in-between for 1000 ms. The participants were allowed 7000 ms to respond.

During the second PET scan, the participants performed a digit n-back task that measures verbal WM updating. In this task, they were to determine if a currently visible digit was the same as the previous digit (1-back) or the digit that was presented three steps back (3-back). The dependent measure was target accuracy on the 3-back vs. 1-back condition. After participants had read the task instructions and completed one 1-back and one 3-back practice task, the PET session was initiated. During scanning, they completed about 55 min of the 1-back (baseline) task, after which they immediately continued with a period of 25 min performing the 3-back task. Task instructions, informing the participant of which n-back version to perform, were shown at the beginning of each task version for 5000 ms, after which a digit was shown for 1500 ms. The digit was replaced by a fixation point that was visible for 450 ms, and after this the fixation point was once again replaced by a digit. The alternation of fixation points and digits continued until the end of the task. Targets encompassed 45% of the trials (i.e., the current digit and the digit n steps back matched), whereas 55% comprised non-targets. The participants responded by pressing a match button for targets and a non-match button for non-targets.

The study protocol and procedure was virtually identical to those employed by Bäckman *et al*.^19^. The only differences between the studies concerned the duration of the letter- memory and digit n-back tasks performed during the PET scans. Unlike Bäckman *et al*.^19^, the current letter-memory task included a 55-minute baseline phase and a 25-minute test phase, with no return to baseline. In other respects, the letter-memory tasks were identical. The digit n-back task was included in the neuropsychological off-line battery in Bäckman *et al*.^19^, whereas here it was altered (i.e., much longer) and performed during PET scanning to mirror the design of the letter-memory task.

### Imaging methods

We used HRRT-PET (High Resolution Research Tomograph; Siemens Medical Solutions, Knoxville, TN, USA) and [^11^C]raclopride with bolus-plus-infusion^32^ to measure striatal dopamine D2 receptor availability during WM vs. control task performance. Raclopride was prepared from [^11^C]methyl triflate following an established procedure^33^. Its radiochemical purity and specific radioactivity were determined using high-performance liquid chromatography and ultraviolet detection at 214 nm. The aimed magnitude of the bolus was 50% of the total tracer volume^34^, and constant infusion continued until 80 min after bolus injection (Kbol = 80 min). At scan start, the actual bolus injection and infused doses did not differ between groups or scans (Table 3). Emission list-mode data were histogrammed into 3D sinograms in 20 time frames of variable length (8 × 2 min, 4 × 3 min, 2 × 4 min, 1 × 5 min, 1 × 6 min, 1 × 8 min, 3 × 8.3 min), taking declining radioactivity and task timing into account. Before each emission scan, a transmission scan was performed using a ^137^Cs point source. Tissue-attenuation maps were reconstructed using the maximum a posteriori transmission data (MAP-TR) algorithm with segmentation. Scattered events were estimated using the single-scatter simulation algorithm, and randoms were estimated from the block singles with a variance-reduction algorithm. All corrections were applied within the statistical image-reconstruction algorithm^35^.

**Table 3:** Administered radioactivities (MBq; mean ± *SD*) in fast bolus and constant infusion formulations, and *p*-values in *t*-tests across groups and scans.

		Bolus	Infusion	Bolus	Infusion	Bolus	Infusion
		Control group		Training group		*t*-test (groups)	
Letter memory	Pre Training	262 ± 18 (*n* = 9)	252 ± 14	272 ± 31 (*n* = 13)	255 ± 28	0.43	0.77
	Post training	267 ± 19 (*n* = 10)	253 ± 15	280 ± 25 (*n* = 11)	265 ± 25	0.52	0.22
	*t* test (scans)	0.76	0.72	0.88	0.43		
n-back	Pre training	267 ± 36 (*n* = 10)	249 ± 32	262 ± 29 (*n* = 13)	264 ± 25	0.74	0.22
	Post training	265 ± 24 (*n* = 10)	260 ± 17	269 ± 17 (*n* = 13)	264 ± 16	0.44	0.51
	*t* test (scans)	0.93	0.43	0.48	0.96		

Special attention was paid to minimization of head-motion artifacts based on earlier observations of its detrimental effects on the PET signal and its interpretation^36^. Specifically, head motion was minimized using an individually molded thermoplastic mask, and image reconstructions were made using an in-house version of the multiple-acquisition frame (MAF)^36^ based motion-compensated image reconstruction algorithm. The algorithm employs external motion tracking (MT) as given by Vicra (Northern Digital) infrared detection, to define motion-free (amplitude less than 2.5 mm) framing of list-mode PET data. This procedure compensates for attenuation map misalignments using mutual information- based image registration and finally combines the motion-free subframes into original, desired framing. In a phantom study by Johansson *et al*. [in preparation], we showed good performance of the MAF-based algorithm, but a residual bias was seen in the presence of rapidly oscillating motion. Rapidly oscillating, high amplitude motion is not often observed in human PET scans but, if present, may introduce erroneous perturbations in the PET signal, which in turn could be incorrectly interpreted as activation effects^36, 37^.

Movements that are beyond the capabilities of the motion-compensation algorithm were sought through analysis of the number of subframes, which was identified as an important factor in our previous phantom experiment with regard to the algorithm’s performance [Johansson *et al*., in preparation]. Analysis showed that in 84% (89/106) of the sessions, no additional acquisition frames were generated, indicating subthreshold (2.5 mm) amplitude of motion in all frames. The remaining cases were investigated further through detailed assessment of the time-motion graphs representing three translations and three rotations of the motion-recording target at the forehead at 1 Hz frequency. On the basis of visual inspection of the motion data, five letter-memory scans from four individuals, and three n-back scans from two individuals were deemed unrecoverable using the motion-compensation algorithm, and were thus excluded from analysis. In addition to those excluded due to extensive motion, the letter-memory PET variables of four subjects were missing due to other technical reasons. In one case, the ^11^C-raclopride infusion was started approximately 20 min overdue, in one case the PET data acquisition failed, and two participants were not PET scanned post training. Thus, a total of eight participants were missing from the PET letter-memory analysis, resulting in an *n* of 20. In turn, the n-back PET variables were missing from three subjects, two of whom were not scanned after training, and in one case the data acquisition failed. Hence, altogether five participants were missing from the n-back analysis, resulting in an *n* of 23.

Structural MR imaging was performed for excluding anatomical abnormalities and for anatomical reference of the PET data. T1-weighted MRI data were acquired using a 3T scanner (Philips Ingenuity TF PET/MR) with a 1 mm × 1 mm × 1 mm voxel size covering the whole brain. The T1-weighted MRI data were pre-processed using FreeSurfer (Athinoula A. Martinos Center for Biomedical Imaging, Massachusetts General Hospital, Charlestown, US) to obtain automated region-of-interest (ROI) definitions^38^. Subsequently, a PET sum image was registered to the skull-stripped MR image, using the mutual-information optimization algorithm in Statistical Parametric Mapping (version 8, SPM8; Wellcome Institute, London, UK). Spatial normalization parameters to the Montreal Neurological Institute (MNI) space coordinates were determined on the basis of MRI data using the unified-segmentation algorithm in SPM8^39^ yielding a mapping for registered PET data normalization. The PET-image analysis was restricted to striatum, which serves as a central hub of dopaminergic activation. Striatal subvolumes were defined on the basis of FreeSurfer segmentations, combining segments of caudate, putamen, and nucleus accumbens into one composite striatal structure in each subject’s individual space. Striatal masks were used in edge-preserving spatial smoothing of the PET data; outside-mask voxels were omitted in the Gaussian filter kernel (10 mm FWHM) calculation, attenuating the impact of partial-volume effects near the edges. Extensive spatial smoothing was deemed necessary for successful calculation of binding potential (BP) from the PET activation data.

Modeling of the dynamic PET data was based on an extension of the conventional simplified reference tissue model (SRTM)^40^, including the effect of activation as an additional parameter. A similar approach as described by Alpert and colleagues^41^ was adopted, where the activation effect on the ^11^C-raclopride signal was modeled by the time-dependent activation function *h*(*t*), and the relevant linear equations are:

$$[\begin{array}{c}{C}_{T}({t}_{1})\\ \vdots \\ {C}_{T}({t}_{m})\end{array}]=[\begin{array}{c}{C}_{R}({t}_{1})\,\\ \vdots \\ {C}_{R}({t}_{m})\end{array}\begin{array}{c}\,{\int }_{0}^{{t}_{1}}{C}_{R}(u)du\,\\ \vdots \\ \,{\int }_{0}^{{t}_{m}}{C}_{R}(u)du\,\end{array}\begin{array}{c}\,-{\int }_{0}^{{t}_{1}}{C}_{T}(u)du\,\\ \vdots \\ \,-{\int }_{0}^{{t}_{m}}{C}_{T}(u)du\end{array}\begin{array}{c}\,-{\int }_{0}^{{t}_{1}}{C}_{T}(u)h(u)du\\ \vdots \\ \,-{\int }_{0}^{{t}_{m}}{C}_{T}(u)h(u)du\end{array}]\,[\begin{array}{c}\begin{array}{c}{R}_{1}\\ {k}_{2}\end{array}\\ {k}_{2a}\\ \gamma \end{array}]$$

where the weighting factor $\,\gamma$ is depicted as the activation effect, ${C}_{T}({t}_{i})$ and ${C}_{R}({t}_{i})$ are the instantaneously measured radioactivity concentrations in target and reference tissue, respectively, and ${R}_{1}$, ${k}_{2}$, ${k}_{2a}$ are the conventional SRTM rate constants. Compared to our previous work^19^, we now employed an explicit BP calculation algorithm for the activation phase, to quantitatively assess the magnitude of this effect relative to baseline. First, baseline BP (BP_0_) was determined from the fit parameters ${k}_{2}$, and ${k}_{2a}$, which in turn were estimated using a linear least-squared solver in Matlab (version R2011b, Mathworks, US). Second, the activation BP (BP_1_) was determined from the fit parameters ${k}_{2}$, ${k}_{2a}$, and $\gamma$, using an integral of $\gamma h(t)$ and averaged over the activation period. As the activation function, we employed a simplified gamma-variate function^42^, with a fixed peak time of 8 min and 20 sec from active task initiation, λ = 3, and onset of activation at 55 min from the injection. Model calculation was restricted to the aforementioned striatal volume, and the resulting parametric images were normalized using the MRI-based deformation into MNI space coordinates. Voxel-wise statistical analysis was performed in SPM8 within a striatal search volume (2934 voxels, ~10 cm^3^), determined as the intersection of individual striatal subvolumes warped in MNI space. Because the images could not be assumed to be normally distributed, non-parametric analyses were conducted using SnPM (version SnPM12, http://www2.warwick.ac.uk/fac/sci/statistics/staff/academic-research/nichols/software/snpm.

A 2 (group) × 2 (time) ANOVA was conducted on BP = BP_1_−BP_0_. Variance smoothing was used with an FWHM of 10 mm. Five thousand permutations were used to determine a *p* < 0.05 threshold within the striatal search space. Paired *t*-tests with a *p* < 0.01 threshold were used to assess main effects of task (letter memory and n-back) on BP, combining both groups.

### Off-line transfer tasks

The pre- and post-training neuropsychological assessments included three WAIS-III subtests (digit span, letter-number sequencing, and digit symbol), a number-letter task, a Simon task, a visuospatial n-back task, and an episodic recall task. A pattern-comparison task, a number-copying task, Trail Making parts A and B, and the WAIS-III vocabulary subtest were only included before training. The pre-training assessment also included a background questionnaire, the Edinburgh Handedness Inventory, and the BDI-II.

#### Digit span

The digit span test was used to assess auditory attention as well as passive (forward span) and active (backward span) WM. Participants were asked to recall sequences of digits in the same or reversed order in which they were presented. Test administration, items, and scoring followed standard WAIS-III procedures^43^. Maximum forward and backward spans were used as dependent measures.

#### Letter-number sequencing

The letter-number sequencing test was also used to assess auditory attention as well as passive and active working memory. Participants were instructed to recall and sort number-letter sequences. Test administration, items, and scoring followed standard WAIS-III procedures^43^. The total score was used as the dependent measure.

#### Visuospatial n-back

The visuospatial n-back task was used to assess visuospatial WM updating^44^. In this task, participants were asked to determine if a currently visible box was in the same location as the previous box (1-back) or the box that was presented two steps back (2-back). A matrix with eight stimulus locations was used (a 3 × 3 matrix without the middle square). Accuracy was used as the dependent measure.

#### Digit-symbol coding

The digit-symbol coding test was used to assess perceptual speed. Participants were asked to draw, as quickly as possible, different symbols in empty spaces on the basis of digits that were located above each empty space. A time limit of 90 sec was used here, but in all other respects test administration, items, and scoring followed standard WAIS-III procedures^43^. The total score was used as the dependent measure.

#### Number-letter task

The number-letter task was used to assess set shifting. Here, participants were asked to categorize either the number or the letter in a number-letter pair depending on in which of two vertically aligned boxes the number-letter pair appeared. The task included both switch and no-switch trials, from which mixing and switching costs were calculated^44^. The switching cost reflects the temporary cognitive load that is related to a task shift, whereas the mixing cost reflects the cost of maintaining attentional control in a situation where two task sets are active. The mixing and switching costs for RTs and accuracy were used as dependent measures.

#### Simon task

The Simon task was used to assess inhibitory control. In this task, participants were to determine the color of a square while disregarding where the square appeared^45^. On congruent trials, the color and location of the square matched the positioning of the appropriate response key, whereas the stimulus and correct response key were crossed on incongruent trials. The Simon effect was calculated by comparing the performance on congruent and incongruent trials. Simon effects for accuracy and RT were used as dependent measures.

#### Episodic word recall

The word recall task was used to assess verbal episodic memory^46^. Participants were instructed to read and remember 18 concrete nouns presented on a computer screen. They were instructed to recall as many words as possible, and the test leader recited any words that had been omitted. After hearing the omitted words, participants again attempted to recall as many words as possible, and once again the test leader recited any omissions. Finally, participants were again asked to recall as many words as possible. The total number of correctly recalled words was used as the dependent measure.

#### Pattern comparison

The pattern-comparison task was used to assess motor speed^47^. In this task, participants were to determine, as quickly as possible, if two symbols were identical. The total number of correctly evaluated symbol-pairs was used as the dependent measure.

#### Number copying

The number-copying task was also used to assess motor speed^47^. Here, participants were asked to copy, as quickly as possible, a set of numbers. The total number of correctly copied numbers was used as the dependent measure.

#### Trail Making

This task was used to assess visual search, hand-eye coordination, and set shifting^48^. In part A, participants were to connect, as quickly as possible, a set of numbers in ascending order. In part B, they were to connect numbers and letters in both ascending and alphabetical order, constantly switching between the two types of stimuli (1-A-2-B, etc.). The dependent variable was the difference score obtained by subtracting the completion time of part A from part B.

#### Vocabulary

The vocabulary test was used to assess word comprehension. Participants were asked to explain the meaning of words of increasing complexity. Test administration, items, and scoring followed standard WAIS-III procedures^43^. The total raw score was used as the dependent measure.

### Statistical analyses

Those participants who were extreme outliers (i.e., showing a deviation of at least three times the interquartile range) before training were removed from the specific statistical analyses on which they deviated. Training effects were analyzed by mixed-model ANOVAs.
